# T-Helper Type 9 Cells Play a Central Role in the Pathogenesis of Respiratory Epithelial Adenomatoid Hamartoma

**DOI:** 10.1097/MD.0000000000001050

**Published:** 2015-07-02

**Authors:** Zhao Wei Gu, Yun Xiu Wang, Zhi Wei Cao

**Affiliations:** From the Department of Otorhinolaryngology, Shengjing Hospital, China Medical University, Shenyang City, Liaoning Province, China (ZWG, ZWC) and Hematological Laboratory, Shengjing Hospital, China Medical University, Shenyang City, Liaoning Province, China (YXW).

## Abstract

The etiology and pathogenesis of respiratory epithelial adenomatoid hamartoma (REAH) remain poorly understood, although some reports have suggested that REAH features an inflammatory process. T-helper type 9 (Th9) cells are a newly identified subset of CD4^+^ T-helper cells characterized by the expression of high levels of interleukin (IL)-9, which may promote inflammation. As REAH may involve an inflammatory process, we evaluated whether IL-9 and/or Th9 cells were present in REAH and compared the levels thereof to those of normal nasal mucosa. Eleven patients with REAH and 5 exhibiting cerebrospinal fluid leakage were included in the study. Flow cytometry was used to measure Th9 cell numbers, a cytometric bead assay was applied to measure IL-9 levels, and real-time polymerase chain reaction was used to quantify the levels of mRNA encoding IL-9. Th9 cells, *IL-9* mRNA, and IL-9 were detected in all REAH and control samples. The proportion of Th9 cells in the patients with REAH was significantly greater than that in the controls. The expression levels of IL-9-encoding mRNA and IL-9 protein were significantly higher in the patients with REAH than in the controls. The Th9 cell subset was expanded, the synthesis of IL-9-encoding mRNA was upregulated, and IL-9 secretion was increased in REAH tissue, suggesting that Th9 cells play a central role in the pathogenesis of the disease.

## INTRODUCTION

Respiratory epithelial adenomatoid hamartoma (REAH) is an uncommon lesion first described by Wenig and Heffner in 1995 and defined as a tumor originating from the surface epithelium, with glandular elements proliferating from this epithelium (and therefore not from seromucous glands).^1^ To date, about 100 incidences have been reported, most as case reports.^2,3^ REAH usually develops in the olfactory cleft (OC).^4–6^ Before 2006, no case of this uncommon lesion originating in the OC had been described, but since then many articles focusing on REAH of the OC have appeared.^5^ Radiologically, REAH in the OC is characterized by soft tissue masses on one or both sides of the OC, and some reports have described significant increases in the width of the OC in such cases.^7^ The current high REAH detection rate is explained by the fact that the OC is now systematically checked on sinonasal computed tomography (CT) scans and during endoscopic surgery; surgical specimens removed from the OC are subjected to pathological processing, and many experienced pathologists now have excellent knowledge of the histological features of this tumor.^5^

The etiology and pathogenesis of REAH remain unclear, although some reports suggest that it features an inflammatory process as the tumor may be complicated by rhinosinusitis and inflammatory polyposis.^1,8,9^ Ozolek and Hunt^10^ found a high-level loss of heterozygosity of loci located on chromosomes 9p and 18q, and an intermediate fractional allelic loss of 31%, in REAH lesions. The cited authors concluded that REAH might thus be a benign neoplasm rather than a hamartoma.^10^

More recently, a novel independent Th-cell subset, characterized by the expression of high levels of interleukin (IL)-9, has been recognized—the T-helper type 9 (Th9) subset.^11–13^ IL-9 affects both inflammatory and normal tissue cells, increasing the numbers of lymphocytes, eosinophils, and mast cells; stimulating IgE secretion; enhancing the responses of mast cells to allergens; promoting mucin expression; and stimulating cytokine secretion by inflammatory cells.^14–17^ Th9 cells have been reported to be involved in various forms of inflammation,^18^ but they have not been sought in REAH. As REAH may involve an inflammatory process, we speculated that IL-9 or Th9 cells might play a role in the condition.

The principal objective of the present study was to determine whether IL-9 or Th9 cells were present in REAH of the OC and to compare the levels of IL-9 and Th9 cells between REAH tissue and normal nasal mucosa.

## MATERIALS AND METHODS

This study was approved by the Shengjing Hospital Institutional Review Board affiliated with China Medical University, and all patients gave signed informed consent.

For 1520 sinus surgeries performed between July 2013 and January 2014, all patients’ data were thoroughly checked for the presence of REAH. The symptoms were nonspecific, and all patients underwent sinus CT preoperatively. Such imaging occasionally revealed soft tissue masses at one or both sides of the OC, raising suspicion of REAH in 16 patients. One patient of allergic rhinitis and one of asthma were excluded. At surgery, masses that were obviously edematous, with a mucosa of indurate texture located in the OC, were sampled and sent for pathological examination. Three patients of nasal polyps were excluded. Control tissues were obtained from the uncinate or ethmoid sinuses of 5 patients exhibiting cerebrospinal fluid leakage but lacking any evidence of sinus disease. No patient took steroids, nonsteroidal antiinflammatory drugs, antihistamines, or macrolide antibiotics for 4 weeks before biopsy. Each tissue sample was divided into 2 parts, 1 of which weighed 30 mg; this aliquot was added to 500 μL of saline to form a single-cell suspension. After centrifugation, the supernatant was stored at −80°C for the measurement of cytokine levels. The Th9 cell number in each precipitate was determined. The other part was immediately frozen in liquid nitrogen and stored at −80°C for RNA extraction.

### RNA Extraction/Reverse Transcription and Real-Time Polymerase Chain Reaction (PCR)

Total RNA was extracted using TRIzol reagent (Invitrogen, Shanghai, China) and 0.5 μg of the total RNA was reverse-transcribed to complementary DNA using a PrimeScript RT kit (Takara, Dalian, China), according to the manufacturer's protocol. Real-time PCR was performed with the aid of an ABI 7500 Real-time PCR System (Applied Biosystems, Foster City, CA) using SYBR Premix Ex Taq (Takara).

The gene encoding glyceraldehyde-3-phosphate dehydrogenase (*GAPDH*; a housekeeping gene) served as a control; *IL-9* was the target gene. The primer sequences used were: forward primer 5′-gaaggtgaaggtcggagtc-3′ and reverse primer 5′-gaagatggtgatgggatttc-3′ for *GAPDH*; and forward primer 5′-ccatggtccttacctctgcc-3′ and reverse primer 5′-agctggatcttcctgcatctt-3′ for *IL-9*.

mRNA levels were measured using the cycle threshold (2^–ΔΔCT^) method and normalized to those of *GAPDH*. A no-template sample served as a negative control.

### Flow Cytometric Analysis of Th9 Cell Numbers

Cells were stimulated with 50 ng/mL phorbol myristate acetate (Sigma–Aldrich, St. Louis, MO), 1 μg/mL ionomycin (Sigma–Aldrich), and 10 μg/mL GolgiStop (BD Biosciences, San Jose, CA) at 37°C under 5% (v/v) CO_2_ for 6 h then stained with fluorescein isothiocyanate-labeled anti-CD4 antibodies (BD Biosciences) and fixed and permeabilized using fix/perm solution (eBioscience, San Diego, CA) according to the manufacturer's instructions. The cells were then incubated with phycoerythrin (PE)-labeled anti-IL-9 antibodies (BD Biosciences) and analyzed on a BD Canto II flow cytometer (BD Biosciences); these data were evaluated using FlowJo software 7.6 (TreeStar Inc., San Carlos, CA).

### Measurement of the IL-9 Concentration

The level of IL-9 in each supernatant was measured using a Cytometric Bead Array (CBA) Flex Set (BD Biosciences) according to the manufacturer's instructions. Briefly, 6 capture bead populations with distinct fluorescence intensities and coated with cytokine-specific capture antibodies were mixed together in equal volumes: 50 μL of each sample and 50 μL of PE-conjugated detection antibodies were added to 50 μL of the mixed-bead populations. Each mixture was incubated for 3 h at room temperature in the dark to form sandwich complexes. Then, the beads were washed with wash buffer, and data acquired with a BD Canto II flow cytometer (BD Biosciences). FACSDiva and BD CBA Software 4.2 (BD Biosciences) were used for the analyses. The limit of detection was 3.1 pg/mL; zero values were assigned when levels were under this limit.

### Statistical Analysis

A statistical analysis was performed using SPSS software ver. 13.0 (SPSS Inc., Chicago, IL). All data are expressed as means ± SEM. The significance levels of between-group differences were determined using the Mann–Whitney *U* test for nonparametric data. A difference was considered statistically significant at *P* < 0.05.

## RESULTS

**FIGURE 1:**
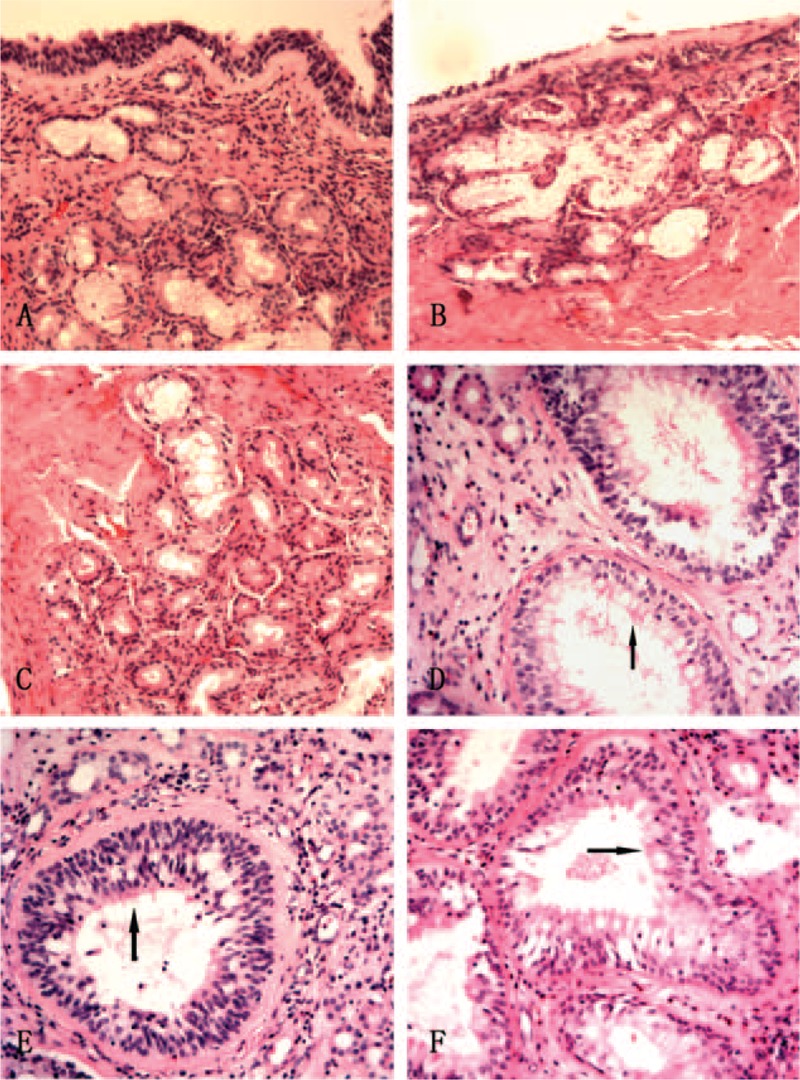
Pathological examination of controls and patients with REAH. (A–C) The pseudostratified ciliated columnar epithelium, serous glands, and mucous glands of the controls. (D–F) In patients with REAH, proliferated glands of various sizes were separated by stromal tissue and lined with a ciliated respiratory epithelium (black arrow). REAH, respiratory epithelial adenomatoid hamartoma (hematoxylin–eosin stain, ×10).

Eleven patients of REAH were diagnosed by the pathologist. Histopathological examinations revealed that the lesions were covered with a ciliated columnar epithelium and that the subepithelial glands had proliferated. The glands were lined by a ciliated respiratory epithelium, and were round or oval in shape, of various sizes, being separated by stromal tissue (Figure 1).

### *IL-9* mRNA Expression

**FIGURE 2:**
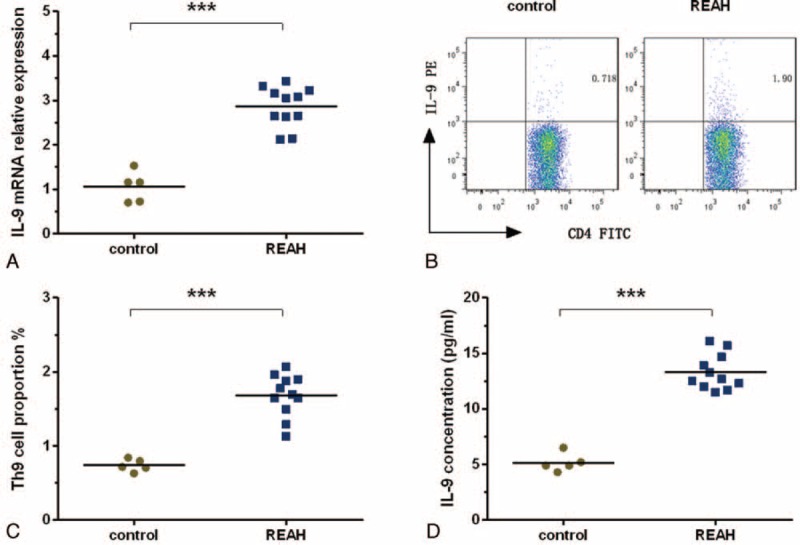
Analysis of *IL-9* mRNA levels, the proportions of Th9 cells, and IL-9 concentrations in controls and patients with REAH. (A) The *IL-9* mRNA levels in control patients and patients with REAH as measured via real-time PCR. (B and C) The proportions of Th9 cells in control patients and patients with REAH as measured using flow cytometry; the numbers in the upper right quadrant are the percentages of IL-9^+^ cells among CD4^+^ cells in gated populations of CD4^+^ T-cells. (D) IL-9 concentrations in controls and patients with REAH as measured using a CBA. FITC = fluorescein isothiocyanate, IL = interleukin, PCR = polymerase chain reaction, PE = phycoerythrin, REAH = respiratory epithelial adenomatoid hamartoma, Th9 = T-helper type 9. ^∗∗∗^*P* < 0.001.

Real-time PCR was used to determine the *IL-9* mRNA expression levels in patients with REAH and controls. *IL-9* mRNA was detected in all samples. According to the Mann–Whitney *U* test, the expression level of *IL-9* mRNA was significantly higher in the patients (2.86 ± 0.45 μg/mL) than in the controls (1.05 ± 0.35 μg/mL; *P* < 0.001; Figure 2A).

### Th9 Cell Proportions

Representative scans showing Th9 cell proportions (CD4^+^IL-9^+^/CD4^+^ T-cells) are shown in Figure 2B. Patients with REAH had significantly higher Th9 cell proportions (1.68 ± 0.29%) than did the controls (0.74 ± 0.08%; *P* < 0.001; Figure 2C).

### IL-9 Concentrations

In agreement with the flow cytometric data on the Th9 cell proportions and real-time PCR analysis of *IL-9* mRNA levels, IL-9 was also upregulated in patients with REAH (13.30 ± 1.59 pg/mL) compared with controls (5.20 ± 0.82 pg/mL; *P* < 0.001; Figure 2D).

## DISCUSSION

REAH, a rare type of hamartoma of the nasal cavity, paranasal sinuses, and nasopharynx, can present as either an isolated lesion (REAHi) or in association with an adjacent inflammatory process such as sinonasal polyposis (REAHsnp).^19^ REAHsnp is difficult to distinguish from nasal polyps alone, and REAHi can mimic several other polypoid masses. In our study, all patients had REAHsnp.

REAH, which has been considered rare, is in fact underdiagnosed. First, it has particular gross characteristics, including a mucosa that is more indurate in texture than that of polyps and a broader basement, but these differences may be ignored in a clinical setting. REAH commonly co-occurs with polyps, and, during surgery, polyp rather than REAH tissue is usually sampled for pathological examination. The diagnosis is thus usually nasal polyps, not REAH. Second, REAH is generally rare in practice, rendering clinicians and pathologists unable to readily identify the condition.^20^

REAH can be diagnosed via a systematic checkup of the OC on a sinonasal CT and during endoscopic surgery, and via careful analysis of surgical specimens removed from the OC for pathological processing.^5^ As REAH comes to be more recognized, larger patient series will accumulate, allowing the etiology and pathogenesis of the disease to be better understood.

The etiologies of hamartomas remain unknown, although several hypotheses have been proposed. One theory suggests that REAH is a benign neoplasm rather than a hamartoma because of the mean fractional allelic loss of 31%,^10^ while another suggests that it may feature an inflammatory process because it may be complicated by rhinosinusitis and inflammatory polyposis.^1,8,9^

In our previous small series,^20^ we found that all 3 patients were associated with nasal polyposis; moreover, 1 patient was associated with fungal sinusitis. Although no direct relationship between fungal sinusitis and REAH is known, the inflammation was serious. In 1 patient, lesions were located bilaterally in the OC and featured obvious edema of the mucosa, which was indurate in texture. The lesions did not appear to be typical polypoid changes, suggesting that inflammation triggered REAH. Of all of our 11 patients, REAH was associated with nasal polyposis, supporting the idea that it may develop secondarily to an inflammatory process.

More recently, a novel independent Th-cell subset, characterized by the expression of high levels of IL-9, has been recognized—the “Th9” subset.^11–13^ Th9 cells are involved in various forms of inflammation.^18^ As REAH may involve an inflammatory process, we explored whether REAH tissue contains Th9 cells. We found, for the first time, that REAH tissue contained Th9 cells and that the Th9 cell proportions were significantly elevated in patients with REAH compared with controls, indicating that such cells may be involved in its pathogenesis.

The *IL-9* gene maps to the long arm of chromosome 5 (5q31–35), which also contains genes encoding IL-4, IL-5, and IL-13.^21^ In allergic asthma, polymorphisms in this region are significantly associated with the development of atopy, bronchial hyperresponsiveness, and elevated total IgE levels.^22–24^

Recent studies have shown that IL-9 promotes inflammation principally by recruiting macrophages, mast cells, and eosinophils. IL-9 is a mast cell growth-enhancing factor.^25^ IL-9 also stimulates mast cell differentiation; mast cells express specific receptors for IL-9.^26,27^ Moreover, IL-9 expression levels correlate with the extent of mast cell infiltration of nasal tissues.^28^ Activated mast cells may represent an alternative source of IL-9, apart from T-cells.^29^

No study has explored IL-9 expression in REAH, but it is associated with the recruitment of large numbers of tryptase-producing mast cells, with no significant difference evident between REAHi and REAHsnp, suggesting that mast cells play an important role in development of this lesion.^4^

In line with the enhanced Th9 cell numbers, *IL-9* mRNA and IL-9 protein levels were significantly higher in REAH tissues. IL-9 enhances mast cell growth; recruitment of large numbers of mast cells in REAH is thus not surprising.^4^

Some studies suggest that IL-9 promotes the recruitment of inflammatory cells to the mucosa of glands, causing inflammatory or epithelial cells to release various immunoglobulins,^15^ in turn increasing the local inflammatory state long-term. Larger lesions trigger obvious clinical symptoms.

We found that Th9 cells and IL-9 were involved in the pathogenesis of REAH, further confirming that the inflammatory response may play a central role in its development, thus negating the theory that REAH is a benign neoplasm.

While Picciotti et al^30^ proposed that REAH induces inflammation, Th9 cells are not the only source of IL-9, and other cells have been reported to produce small amounts of IL-9 with stimulation or under special circumstances,^31^ including natural killer T-cells and type 2 innate lymphoid cells. Further work is needed to determine the amounts of IL-9 that these cell types produce, if it indeed plays a role in REAH. In addition, REAHi requires further research. Does the inflammation disappear after REAH formation, and is its development thus independent of inflammation? The levels of Th9 cells and IL-9 in REAHi require assessment. The results will play a crucial role in further elucidating the pathogenesis of REAH.

## CONCLUSIONS

The Th9 cell subset was expanded, synthesis of IL-9-encoding mRNA upregulated, and IL-9 secretion was increased in REAH, suggesting that Th9 cells play a central role in its pathogenesis.

## References

[1] Wenig BM Heffner DK Respiratory epithelial adenomatoid hamartomas of the sinonasal tract and nasopharynx: a clinicopathologic study of 31 cases. *Ann Otol Rhinol Laryngol* 1995; 104:639–645. 7639474 10.1177/000348949510400809

[2] Eloy JA Friedel ME Eloy JD Bilateral olfactory fossa respiratory epithelial adenomatoid hamartomas. *Arch Otolaryngol Head Neck Surg* 2011; 137:820–822. 21844417 10.1001/archotol.137.8.820

[3] Gu Z Cao Z Frontal sinus pneumocele associated with respiratory epithelial adenomatoid hamartoma and nasal polyps. *Otolaryngol Head Neck Surg* 2012; 147:177–178. 22228600 10.1177/0194599811432232

[4] Gauchotte G Marie B Gallet P Respiratory epithelial adenomatoid hamartoma: a poorly recognized entity with mast cell recruitment and frequently associated with nasal polyposis. *Am J Surg Pathol* 2013; 37:1678–1685. 24121171 10.1097/PAS.0000000000000092

[5] Nguyen DT Gauchotte G Arous F Respiratory epithelial adenomatoid hamartoma of the nose: an updated review. *Am J Rhinol Allergy* 2014; 28:187–192. 25198016 10.2500/ajra.2014.28.4085

[6] Vira D Bhuta S Wang MB Respiratory epithelial adenomatoid hamartomas. *Laryngoscope* 2011; 121:2706–2709. 22006652 10.1002/lary.22399

[7] Lima NB Jankowski R Georgel T Respiratory adenomatoid hamartoma must be suspected on CT-scan enlargement of the olfactory clefts. *Rhinology* 2006; 44:264–269. 17216743

[8] Delbrouck C Fernandez Aguilar S Choufani G Respiratory epithelial adenomatoid hamartoma associated with nasal polyposis. *Am J Otolaryngol* 2004; 25:282–284. 15239039 10.1016/j.amjoto.2004.02.005

[9] Liang J O’Malley BW Jr Feldman M A case of respiratory epithelial adenomatoid hamartoma. *Am J Otolaryngol* 2007; 28:277–279. 17606048 10.1016/j.amjoto.2006.09.013

[10] Ozolek JA Hunt JL Tumor suppressor gene alterations in respiratory epithelial adenomatoid hamartoma (REAH): comparison to sinonasal adenocarcinoma and inflamed sinonasal mucosa. *Am J Surg Pathol* 2006; 30:1576–1580. 17122514 10.1097/01.pas.0000213344.55605.77

[11] Cortelazzi C Campanini N Ricci R Inflammed skin harbours Th9 cells. *Acta Derm Venereol* 2013; 93:183–185. 22855259 10.2340/00015555-1408

[12] Schlapbach C Gehad A Yang C Human TH9 cells are skin-tropic and have autocrine and paracrine proinflammatory capacity. *Sci Transl Med* 2014; 6:219ra8. 10.1126/scitranslmed.3007828 PMC4102325 24431112

[13] Ma L Xue HB Guan XH Possible pathogenic role of T helper type 9 cells and interleukin (IL)-9 in atopic dermatitis. *Clin Exp Immunol* 2014; 175:25–31. 24032555 10.1111/cei.12198 PMC3898551

[14] McLane MP Haczku A van de Rijn M Interleukin-9 promotes allergen-induced eosinophilic inflammation and airway hyperresponsiveness in transgenic mice. *Am J Respir Cell Mol Biol* 1998; 19:713–720. 9806735 10.1165/ajrcmb.19.5.3457

[15] Dugas B Renauld JC Pène J Interleukin-9 potentiates the interleukin-4-induced immunoglobulin (IgG, IgM and IgE) production by normal human B lymphocytes. *Eur J Immunol* 1993; 23:1687–1692. 7686859 10.1002/eji.1830230743

[16] Louahed J Toda M Jen J Interleukin-9 upregulates mucus expression in the airways. *Am J Respir Cell Mol Biol* 2000; 22:649–656. 10837360 10.1165/ajrcmb.22.6.3927

[17] Soussi-Gounni A Kontolemos M Hamid Q Role of IL-9 in the pathophysiology of allergic diseases. *J Allergy Clin Immunol* 2001; 107:575–582. 11295641 10.1067/mai.2001.114238

[18] Kaplan MH Th9 cells: differentiation and disease. *Immunol Rev* 2013; 252:104–115. 23405898 10.1111/imr.12028 PMC3982928

[19] Hawley KA Pabon S Hoschar AP The presentation and clinical significance of sinonasal respiratory epithelial adenomatoid hamartoma (REAH). *Int Forum Allergy Rhinol* 2013; 3:248–253. 23038055 10.1002/alr.21083

[20] Cao Z Gu Z Yang J Respiratory epithelial adenomatoid hamartoma of bilateral olfactory clefts associated with nasal polyposis: three cases report and literature review. *Auris Nasus Larynx* 2010; 37:352–356. 19942387 10.1016/j.anl.2009.10.003

[21] van Leeuwen BH Martinson ME Webb GC Molecular organization of the cytokine gene cluster, involving the human IL-3, IL-4, IL-5, and GM-CSF genes, on human chromosome 5. *Blood* 1989; 73:1142–1148. 2564789

[22] Postma DS Bleecker ER Amelung PJ Genetic susceptibility to asthma–bronchial hyperresponsiveness coinherited with a major gene for atopy. *N Engl J Med* 1995; 333:894–900. 7666875 10.1056/NEJM199510053331402

[23] Noguchi E Shibasaki M Arinami T Evidence for linkage between asthma/atopy in childhood and chromosome 5q31–q33 in a Japanese population. *Am J Respir Crit Care Med* 1997; 156:1390–1393. 9372650 10.1164/ajrccm.156.5.9702084

[24] Doull IJ Lawrence S Watson M Allelic association of gene markers on chromosomes 5q and 11q with atopy and bronchial hyperresponsiveness. *Am J Respir Crit Care Med* 1996; 153:1280–1284. 8616554 10.1164/ajrccm.153.4.8616554

[25] Hültner L Druez C Moeller J Mast cell growth-enhancing activity (MEA) is structurally related and functionally identical to the novel mouse T cell growth factor P40/TCGFIII (interleukin 9). *Eur J Immunol* 1990; 20:1413–1416. 2115002 10.1002/eji.1830200632

[26] Godfraind C Louahed J Faulkner H Intraepithelial infiltration by mast cells with both connective tissue-type and mucosal-type characteristics in gut, trachea, and kidneys of IL-9 transgenic mice. *J Immunol* 1998; 160:3989–3996. 9558107

[27] Druez C Coulie P Uyttenhove C Functional and biochemical characterization of mouse P40/IL-9 receptors. *J Immunol* 1990; 145:2494–2499. 2145361

[28] Nouri-Aria KT Pilette C Jacobson MR IL-9 and c-Kit+ mast cells in allergic rhinitis during seasonal allergen exposure: effect of immunotherapy. *J Allergy Clin Immunol* 2005; 116:73–79. 15990777 10.1016/j.jaci.2005.03.011

[29] Stassen M Müller C Arnold M IL-9 and IL-13 production by activated mast cells is strongly enhanced in the presence of lipopolysaccharide: NF-kappa B is decisively involved in the expression of IL-9. *J Immunol* 2001; 166:4391–4398. 11254693 10.4049/jimmunol.166.7.4391

[30] Picciotti PM Calò L Mulè A Rhino sinusal bilateral hamartoma: a case report. *Auris Nasus Larynx* 2008; 35:569–571. 18207343 10.1016/j.anl.2007.10.006

[31] Kaplan MH Hufford MM Olson MR The development and in vivo function of T helper 9 cells. *Nat Rev Immunol* 2015; 15:295–307. 25848755 10.1038/nri3824 PMC4445728

